# Meta-analysis of Hsa-mir-499 polymorphism (rs3746444) for cancer risk: evidence from 31 case-control studies

**DOI:** 10.1186/s12881-014-0126-1

**Published:** 2014-11-30

**Authors:** Chen Chen, Shenglan Yang, Sandip Chaugai, Yan Wang, Dao Wen Wang

**Affiliations:** Department of Internal Medicine and the Institute of Hypertension, Tongji Hospital, Tongji Medical College of Huazhong University of Science and Technology, 1095# Jiefang Ave, Wuhan, 430030 People’s Republic of China; Department of Internal Medicine, The First Affiliated Hospital of Chongqing Medical University, Chongqing, China

**Keywords:** miRNA polymorphism, Cancer risk, Asian, rs3746444, Meta-analysis

## Abstract

**Background:**

MicroRNAs (miRNAs) are a family of endogenous, small and non-coding RNAs that regulate gene expression negatively at the post-transcriptional level by suppressing translation or degrading target mRNAs, and are involved in diverse biological and pathological processes. Single nucleotide polymorphisms (SNPs) which are located in the miRNA-coding genes may participate in the process of development and diseases by altering the expression of mature miRNA. Recent studies investigating the association between hsa-mir-499 polymorphism (rs3746444) and cancer risk have yielded conflicting results.

**Methods:**

In this meta-analysis, we conducted a search of case–control studies on the associations of SNP rs3746444 with susceptibility to cancer in electronic databases. A total of 31 studies involving 12799 cases and 14507 controls were retrieved and the strength of the association was estimated by pooled odds ratios (ORs) and 95% confidence intervals (CIs). Hardy-Weinberg equilibrium (HWE) was assessed by the goodness-of-fit chi-square test in controls. Subgroup analyses were done by racial descent and cancer type. Publication bias of literatures was evaluated by visual inspection of funnel plots and the linear regression asymmetry test by Egger et al. Sensitivity analysis was conducted by excluding one study at a time to examine the influence of individual data set on the pooled ORs.

**Results:**

Overall, significant association between rs3746444 polymorphism and susceptibility to cancer was identified in TC versus TT and TC/CC versus TT (dominant) models. In the stratified analyses, increased risks were found in Asians, but not in Caucasians in all comparison models tested. Moreover, significant association with an increased risk was found in Chinese population. Also, much higher significant association with increased cancer risks were found in Iranian population. In different cancer types, a decreased risk was found in esophageal cancer.

**Conclusion:**

Our meta-analysis suggested that hsa-mir-499 rs3746444 T > C polymorphism is associated with the risk of cancer in Asians, mainly in Iranian and Chinese population. However, rs3746444 T > C polymorphism is negatively associated with the risk of esophageal cancer.

**Electronic supplementary material:**

The online version of this article (doi:10.1186/s12881-014-0126-1) contains supplementary material, which is available to authorized users.

## Background

In early 1990s, microRNAs (miRNAs) were first discovered through analysis of developmental timing mutants in *C. elegans* by several groups, simultaneously [1,2]*.* In the last decade, the study of miRNA biology has attracted remarkable attention, resulting in rapid advances, and revealing miRNAs as key gene regulators in diverse biological pathways [3]. miRNAs participate in regulation of stem cell functions [4], development [5], drug resistance [6], metabolism and metabolic disorders [7], cardiovascular [8] and malignant diseases [9,10].

miRNAs are hairpin-derived RNAs ~20-24 nucleotides long, which post-transcriptionally repress the expression of target genes usually by binding to the 3′-untranslated region (3′ UTR) of messenger RNA (mRNA) in a broad range of organisms in both normal physiological and disease contexts [11,12]. The majority of human miRNA loci are located within intronic regions and are transcribed by RNA polymerase II as part of their hosting transcription units [13]. Typically, following RNA polymerase II-mediated transcription, the long primary precursors (pri-miRNAs) are cleaved by the nuclear RNase III (Drosha) to release ~70 nt pre-miRNAs [14]. The resulting transcripts adopt a stem-loop structure and are then exported to the cytoplasm where they are subsequently processed by another RNase III (Dicer) to generate mature double-stranded ~22 nt miRNAs [15]. Subsequently, one strand of this duplex is incorporated into an Argonaute-containing RNA-induced silencing complex (RISC), resulting in the translational repression and/or degradation of their target mRNAs [16].

Recent evidences support that single nucleotide polymorphisms (SNPs), occurring in DNA sequences of miRNA-coding genes or in miRNA-binding site in mRNAs, contribute to the biogenesis and functions of miRNAs [17]. Gain/loss-of-function of miRNA polymorphisms may result in enhancing of the combination of the miRNA to the targets or losing control of the mRNAs, which may be associated with diseases [18].

Reports on the associations between SNPs in pre-miRNA or SNPs in binding sites in 3′ UTR of miRNA targeting mRNAs and human diseases have provided new insights into the molecular mechanisms of pathophysiological processes in humans. rs4846049 (G > T) of MTHFR gene is associated with increased risk for coronary heart diseases, and the potentially pathogenetic mechanism may be SNP-modified posttranscriptional gene regulation by miRNA-149 to MTHFR [19]. MiR-196a binding-site SNP regulates RAP1A expression contributing to esophageal squamous cell carcinoma risk and metastasis [20]. A miRNA-binding SNP (1010A/G) located within 3′-UTR of HOXB5 is associated with gene expression and may be a promising prognostic factor for bladder cancer [21]. The A to G base change of rs999885 may provide a protective effect against chronic HBV infection but an increased risk for HCC in HBV persistent carriers by altering the expression of the miR-106b-25 cluster [22]. miR-200b/200c/429-binding site polymorphism in the 3′-UTR of AP-2α gene is associated with cisplatin resistance, suggesting that SNP (rs1045385) A > C variation may be a potential prognostic marker for cisplatin treatment [23].

To date, many studies explored the association between rs3746444 T > C SNP in hsa-mir-499 and susceptibility to diseases, such as breast cancer [24–26], lung cancer [27], congenital heart disease [28], dilated cardiomyopathy [29], gallbladder cancer [30], squamous cell carcinoma of the head and neck [31], chronic obstructive pulmonary disease [32], liver cancer [33], rheumatoid arthritis [34], coronary artery disease [35], and colorectal cancer [36]. Especially, one study suggested that miR-499 rs3746444*T alleles might be protective for breast cancer [37], but another found that the miR-499 rs3746444 C allele increased cancer risk in the allelic contrast model and in the dominant model, especially in breast cancer [38], while another study showed that no significant associations were observed between rs3746444 in miR-499 and breast cancer susceptibility [39]. Recently, a meta-analysis suggested that polymorphism of hsa-mir-499 rs3746444 T > C was not associated with increased susceptibility to cancers [40,41], while other systematic analysis supported that hsa-mir-499 rs3746444 polymorphism contributed to the susceptibility to cancers [42–45].

The results of these observations remain controversial and inconclusive. In the present study, we conducted a meta-analysis in order to derive more precise and more comprehensive estimation of the associations between the SNP hsa-mir-499 rs3746444 T > C and susceptibility to cancers to quantify the potential between-study heterogeneity.

## Methods

### Study selection

We performed a publication search in PubMed, EMBASE, ISI Web of Science, The Cochrane Library, ScienceDirect, EBSCO, Ovid, Wiley Online Library, and HighWire databases with the following search terms: (miR-499 OR rs3746444) AND (cancer), by two independent investigators (Chen Chen and Shenglan Yang, last search update: June 29, 2014). Hand searches were also performed to identify additional articles in the reference lists of included articles not retrieved by initial electronic search. Publication date and publication language were not restricted in our search. All studies matching the inclusion criteria were retrieved for further examination and data extraction. All of the investigators have received training in literature search, statistics and evidence-based medicine.

### Inclusion and exclusion criteria

Studies included in current meta-analysis had to meet all the following criteria: (1) evaluated the associations between the hsa-mir-499 rs3746444 polymorphism and cancer risk; (2) studied on human beings; (3) diseases were confirmed by histology, imaging or pathology; (4) a case–control design; (5) detailed genotype data were provided for the calculation of odds ratio (OR) and 95% confidence interval (CIs); (6) if serial studies of the same population from the same group were reported, the latest study was included. Studies were excluded when they represented duplicates of previous publications, or were meta -analyses, meeting abstracts, letters, reviews, or editorial articles.

### Data extraction

Two investigators (Chen Chen and Shenglan Yang) independently extracted data from the included studies using a standard protocol and data-collection form according to the inclusion criteria listed above, and reached consensus on all items. Data extracted from eligible studies included the first author’s name, year of publication, country of origin, ethnicity, cancer type, genotyping method, total numbers of cases and controls, and genotype frequencies of cases and controls. For study including subjects of different countries of origin group from same ethnicity, we combined them together. The ethnic descents were categorized as Caucasian or Asian. If different results were generated, the two authors would check the data and have had a discussion to come to an agreement. Two senior investigators (Yan Wang and Dao Wen Wang) were invited to the discussion if disagreement still existed.

### Statistical analysis

For each study, the departure of frequencies of hsa-mir-499 polymorphism from expectation under Hardy-Weinberg equilibrium (HWE) was assessed by the goodness-of-fit chi-square test in controls. *P <* 0.05 was considered representative of a departure from HWE.

OR corresponding to 95% CI was used to assess the strength of association between hsa-miR-499 rs3746444 T > C polymorphism and susceptibility to cancer risk. The significance of the pooled OR was determined by the Z-test, and *P <* 0.05 was considered as statistically significant. Pooled ORs were calculated for allele frequency comparison (C versus T, TC versus TT, CC versus TT, TC/CC versus TT (dominant) and CC versus TC/TT (recessive)), respectively. Subgroup analyses were done by racial descent and cancer type. Statistical heterogeneity among the studies was estimated using chi-square-based Q-test, a *P* value greater than 0.1 indicates no significant heterogeneity and the pooled OR was estimated by the fixed-effects model (the Mantel-Haenszel method); otherwise, the random-effects model (the DerSimonian and Laird method) was employed [46]. Publication bias of literatures was evaluated by funnel plots and the linear regression asymmetry test by Egger et al. [47]. An asymmetric plot suggests a possible publication bias and the *P* value of Egger’s test less than 0.05 was considered representative of statistically significant publication bias.Sensitivity analysis was conducted by deleting one study at a time to examine the influence of individual data set on the pooled ORs.

All of the statistical tests were performed with Review Manage (v.4.2; Oxford, England) and STATA software version 11.0 (STATA Corporation, College Station, TX, USA). All the *P* values are two-sided.

## Results

### Study characteristics

**Figure 1:**
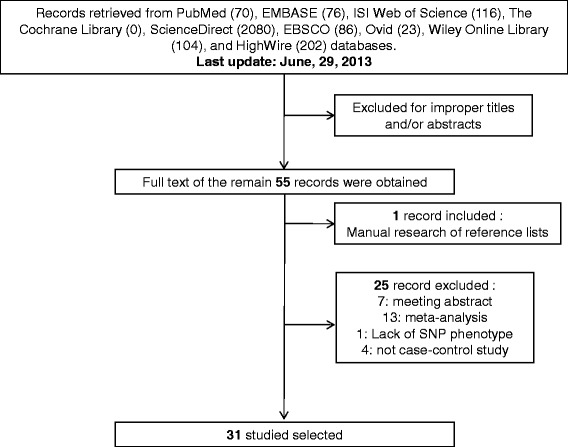
**Flow diagram of study identification.**

After first search in PubMed, EMBASE, ISI Web of Science, The Cochrane Library, ScienceDirect, EBSCO, Ovid, Wiley Online Library, and HighWire databases, 70, 76, 116, 0, 2080, 86, 23, 104, and 202 articles, respectively, were retrieved. Of these, 55 articles were relevant to the search words (Figure 1). After further manual search of reference lists, one more study was included. Then, 25 studies were excluded (7 meeting abstracts, 13 meta-analyses, 1 without detailed allele frequency data, and 4 non-case–control studies) and finally 31 studies involving a total of 12799 disease cases and 14507 controls met the inclusion criteria and were subjected to further examination. Characteristics of included studies are summarized in Table 1, including ethnicity, genotype detection method and cancer type. All studies were case–control studies, including 8 liver cancer studies [33,48–54], 4 breast cancer studies [24–26,55], 4 gastric cancer studies [56–59], 3 colorectal cancer studies [36,60,61], 2 lung cancer studies [27,62], 2 esophageal cancer studies [63,64], and others (1 cervical cancer study [65], 1 bladder cancer study [66], 1 prostate cancer study [67], 1 head and neck cancer study [31], 1 gallbladder cancer study [30], 1 childhood acute lymphoblastic leukemia [68], 1 renal cell carcinoma [69] and 1 oral squamous cell carcinoma [70]). Cancers were histologically or pathologically diagnosed in all studies. There were 28 studies of Asian descents (16 Chinese, 4 Indian, 3 Korean, 2 Iranians and 3 others) and 3 studies of Caucasian descents. Several genotyping methods were employed in the studies including Taqman, polymerase chain reaction-restriction fragment length polymorphism (PCR-RFLP), DNA sequencing, high resolution melting analysis (HRMA), T-ARMS-PCR, MALDI-TOF MS and Sequenom MassARRAY. PRISMA checklist was generated to provide detailed description of this meta-analysis (Additional file 1: Table S1).

**Table 1:** **Characteristics of studies included in the meta-analysis**

	**First Author**	**Year**	**Country**	**Ethnicity**	**Cancer type**	**HWE of control**	**No. (case/control)**	**Genotypic frequencies of cases (n, (%))**			**Allele frequencies of cases (n, (%))**		**Genotypic frequencies of controls (n, (%))**			**Allele frequencies of controls (n, (%))**	
								**TT**	**TC**	**CC**	**T**	**C**	**TT**	**TC**	**CC**	**T**	**C**
1	Hu	2009	China	Asian	Breast cancer	0.057	1009/1093	707 (70)	258 (25.6)	44 (4.4)	1672 (82.9)	346 (17.1)	816 (74.7)	248 (22.7)	29 (2.6)	1880 (86)	306 (14)
2	Tian	2009	China	Asian	Lung cancer	0.404	1058/1035	781 (73.8)	253 (23.9)	24 (2.3)	1815 (85.8)	301 (14.2)	755 (73.0)	254 (24.5)	26 (2.5)	1764 (85.2)	306 (14.8)
3	Catucci	2010	Italy; Germany	Caucasian	Breast cancer	0.284	1579/2167	950 (60.16)	545 (34.52)	84 (5.32)	2445 (77.4)	713 (22.6)	1305 (60.22)	742 (34.24)	120 (5.54)	3352 (77.3)	982 (22.7)
4	Liu	2010	US	Caucasian	Squamous cell carcinoma of the head and neck	0.441	1109/1130	745 (67.2)	309 (27.9)	55 (4.9)	1799 (81.1)	419 (18.9)	710 (62.8)	366 (32.4)	54 (4.8)	1786 (79)	474 (21)
5	Srivastava	2010	India	Asian	Gallbladder cancer	0.566	230/230	112 (48.7)	97 (42.2)	21 (9.1)	321 (69.8)	139 (30.2)	121 (52.6)	94 (40.9)	15 (6.5)	336 (73)	124 (27)
6	Okubo	2010	Japan	Asian	Gastric cancer	0.048	697/552	364 (65.9)	151 (27.4)	37 (6.7)	879 (79.6)	225 (20.4)	466 (66.9)	198 (28.4)	33 (4.7)	1130 (81.1)	264 (18.9)
7	Akkiz	2011	Turkish	Asian	Hepatocellular carcinoma	0.036	222/222	45 (20.3)	87 (39.2)	90 (40.5)	177 (40)	267 (60)	47 (21.2)	93 (41.9)	82 (36.9)	187 (42.1)	257 (57.9)
8	George	2011	India	Asian	Prostate cancer	0.073	159/230	48 (30.2)	98 (61.6)	13 (8.2)	194 (61)	124 (39)	104 (45.2)	92 (40.0)	34 (14.8)	300 (65.2)	160 (34.8)
9	Min	2011	Korea	Asian	Colorectal cancer	0.453	446/502	292 (65.5)	142 (31.8)	12 (2.7)	726 (81.4)	166 (18.6)	334 (66.5)	154 (30.7)	14 (2.8)	822 (81.9)	182 (18.1)
10	Mittal	2011	India	Asian	Bladder cancer	0.029	212/250	95 (44.8)	92 (43.4)	25 (11.8)	282 (66.5)	142 (33.5)	121 (48.4)	94 (37.6)	35 (14.0)	336 (67.2)	164 (32.8)
11	Vinci	2011	Italy	Caucasian	Lung cancer	0.503	101/129	53 (52.5)	41 (40.6)	7 (6.9)	147 (72.8)	55 (27.2)	70 (54.2)	48 (37.2)	11 (8.6)	188 (72.9)	70 (27.1)
12	Zhou	2011	China	Asian	Cervical squamous cell carcinoma	0.005	226/309	134 (59.3)	84 (37.2)	8 (3.5)	352 (77.9)	100 (22.1)	223 (68.2)	71 (23.0)	15 (4.8)	517 (83.7)	101 (16.3)
13	Alshatwi	2012	Saudi	Asian	Breast cancer	0.304	92/89	27 (29.3)	57 (62)	8 (8.7)	111 (60.3)	73 (39.7)	40 (45)	36 (40.4)	13 (14.6)	116 (65.2)	62 (34.8)
14	Kim	2012	Korea	Asian	Hepatocellular carcinoma	0.278	159/201	109 (68.6)	47 (29.5)	3 (1.9)	265 (83.3)	53 (16.7)	120 (59.7)	74 (36.8)	7 (35)	314 (78.1)	88 (21.9)
15	Umar	2012	India	Asian	Esophageal cancer	0.087	289/309	155 (53.6)	122 (42.2)	12 (4.2)	432 (74.7)	146 (25.3)	149 (48.2)	140 (45.3)	20 (6.5)	438 (70.9)	180 (29.1)
16	Xiang	2012	China	Asian	Hepatocellular carcinoma	0.284	100/100	36 (36)	40 (40)	24 (24)	112 (56)	88 (44)	54 (54)	36 (36)	10 (10)	144 (72)	56 (28)
17	Zhou	2012	China	Asian	Hepatocellular carcinoma	0.1	186/483	141 (75.8)	41 (22.0)	4 (2.2)	323 (86.8)	49 (13.2)	371 (76.8)	100 (20.7)	12 (2.48)	842 (87.2)	124 (12.8)
18	Ahn	2012	Korea	Asian	Gastric cancer	0.829	461/477	323 (70.1)	123 (26.7)	15 (3.3)	769 (83.4)	153 (16.6)	299 (66.9)	134 (30.0)	14 (3.1)	732 (81.9)	162 (18.1)
19	Hasani	2013	Iran	Asian	Childhood acute lymphoblastic leukemia	0.249	75/115	35 (46.7)	28 (37.3)	12 (16.0)	98 (65.3)	52 (34.7)	61 (53.0)	42 (36.5)	12 (10.4)	164 (71.3)	66 (28.7)
20	Wei	2013	China	Asian	Esophageal cancer	0.036	380/380	291 (81.3)	60 (16.8)	7 (2.0)	642 (89.7)	74 (10.3)	289 (76.9)	76 (20.2)	11 (2.9)	654 (87)	98 (13)
21	Zou	2013	China	Asian	Hepatocellular carcinoma	0.005	185/185	136 (73.5)	44 (23.8)	5 (2.7)	316 (85.4)	54 (14.6)	123 (66.5)	48 (25.9)	14 (7.6)	294 (79.5)	76 (20.5)
22	Lv	2013	China	Asian	Colorectal cancer	0.082	346/504	258 (74.6)	86 (24.6)	2 (5.8)	602 (87)	90 (13)	366 (72.6)	121 (24.0)	17 (3.4)	853 (84.6)	155 (15.4)
23	Hu	2013	China	Asian	Colorectal cancer	0.162	276/373	157 (74.4)	49 (23.2)	5 (2.4)	363 (86)	59 (14)	282 (75.6)	81 (21.7)	10 (2.7)	645 (86.5)	101 (13.5)
24	Shan	2013	China	Asian	Hepatocellular carcinoma	0.005	172/185	128 (74.4)	37 (21.5)	7 (4.1)	293 (85.2)	51 (14.8)	123 (66.7)	48 (25.8)	14 (7.5)	294 (79.5)	76 (20.5)
25	Wu	2013	China	Asian	Gastric cancer	0.854	200/211	149 (74.5)	47 (23.5)	4 (2.0)	345 (86.3)	55 (13.7)	166 (78.7)	42 (19.9)	3 (1.4)	374 (88.6)	48 (11.4)
26	Omrani	2014	Iran	Asian	Breast cancer	<0.001	236/203	131 (55.5)	44 (18.6)	61 (25.8)	306 (64.8)	166 (35.2)	130 (64.0)	48 (23.7)	25 (12.3)	308 (75.8)	98 (24.2)
27	Du	2014	China	Asian	Renal cell cancer	0.594	354/362	251 (70.9)	94 (26.6)	9 (2.5)	596 (84.2)	112 (15.8)	255 (70.4)	96 (26.5)	11 (3.1)	606 (83.7)	118 (16.3)
28	Huo	2014	China	Asian	Oral squamous cell carcinoma	0.419	872/667	616 (70.6)	243 (27.9)	13 (1.5)	1475 (84.6)	269 (15.4)	505 (75.7)	148 (22.2)	14 (2.1)	1158 (86.8)	176 (13.2)
29	Ma	2014	China	Asian	Hepatocellular carcinoma	<0.001	984/969	724 (73.6)	241 (24.5)	19 (1.9)	1689 (85.8)	279 (14.2)	765 (79.0)	179 (18.4)	25 (2.6)	1709 (88.2)	229 (11.8)
30	Pu	2014	China	Asian	Gastric cancer	0.082	196/504	141 (71.9)	50 (25.5)	5 (2.6)	332 (84.7)	60 (15.3)	366 (72.6)	121 (24.0)	17 (3.4)	853 (84.6)	155 (15.4)
31	Chu	2014	China	Asian	Hepatocellular carcinoma	0.321	188/337	119 (63.30)	60 (31.91)	9 (4.79)	298 (79.3)	78 (20.7)	281 (83.38)	55 (16.32)	1 (0.30)	617 (91.5)	57 (8.5)

### Meta-analysis results

The association between hsa-mir-499 rs3746444 polymorphism and susceptibility to cancer was analyzed in 31 independent studies. The results are shown in Table 2 and Additional file 2: Figures S1, S2 and S3. Significant association between rs3746444 polymorphism and susceptibility to cancer was identified in TC versus TT and TC/CC versus TT (dominant) models, when all the eligible studies were pooled (C versus T: OR = 1.08, 95% CI 0.99-1.17, *P =* 0.08; TC versus TT: OR = 1.11, 95% CI 1.00-1.23, *P =* 0.04; CC versus TT: OR = 1.02, 95% CI 0.85-1.22, *P =* 0.85; TC/CC versus TT (dominant): OR = 1.11, 95% CI 1.01-1.23, *P =* 0.03; CC versus TC/TT (recessive): OR = 0.98, 95% CI 0.81-1.17, *P =* 0.79). Next, subgroup analyses were performed. Twenty-eight out of the thirty-one included studies were conducted in Asian population. In ethnicity subgroup analysis, significantly increased risks were found in Asians (TC versus TT: OR = 1.14; 95% CI = 1.02-1.27, *P =* 0.02; TC/CC versus TT (dominant): OR = 1.14; 95% CI = 1.02-1.27, *P =* 0.02), consistently. This increased risk of cancer was especially significant in Iranian (C versus T: OR = 1.57; 95% CI = 1.23-2.01, *P =* 0.0003; CC versus TT: OR = 2.23; 95% CI = 1.42-3.51, *P =* 0.0005; CC versus TC/TT (recessive): OR = 2.23; 95% CI = 1.44-3.45, *P =* 0.0003) and Chinese population (TC versus TT: OR = 1.18; 95% CI = 1.02-1.35, *P =* 0.02). However, no significant association between hsa-mir-499 rs3746444 T > C polymorphism and cancer risk was found in Caucasians in any of the genetic models tested. Further subgroup analysis based on cancer type revealed a decreased risk for esophageal cancer (C versus T: OR = 0.80; 95% CI = 0.66-0.98, *P =* 0.03).

**Table 2:** **Meta-analysis for the hsa-miR-499 rs3746444 T > C polymorphism and cancer risk**

**Genetic model**	**N** ^**a**^	**C vs T**			**TC vs TT**			**CC vs TT**			**TC/CC vs TT (dominant)**			**CC vs TC/TT (recessive)**		
		**Pooled OR [95% CI]**	***P*** **h** ^**b**^	***P*** ^**c**^	**Pooled OR [95% CI]**	***P*** **h** ^**b**^	***P*** ^**c**^	**Pooled OR [95% CI]**	***P*** **h** ^**b**^	***P*** ^**c**^	**Pooled OR [95% CI]**	***P*** **h** ^**b**^	***P*** ^**c**^	**Pooled OR [95% CI]**	***P*** **h** ^**b**^	***P*** ^**c**^
**Total**	31	1.08 (0.99-1.17)	<0.00001	0.08	1.11 (1.00-1.23)	<0.00001	**0.04**	1.02 (0.85-1.22)	0.002	0.85	1.11 (1.01-1.23)	<0.00001	**0.03**	0.98 (0.81-1.17)	0.001	0.79
**Ethnicities**																
Asian	28	1.10 (1.00-1.21)	<0.00001	0.06	1.14 (1.02-1.27)	<0.00001	**0.02**	1.02 (0.82-1.27)	0.0009	0.86	1.14 (1.02-1.27)	<0.00001	**0.02**	0.97 (0.78-1.20)	0.0004	0.76
Chinese	16	1.11 (0.95-1.28)	<0.00001	0.18	1.18 (1.02-1.35)	0.0002	**0.02**	0.92 (0.65-1.32)	0.001	0.66	1.15 (0.99--1.34)	<0.00001	0.07	0.89 (0.63-1.24)	0.004	0.48
Indian	4	1.03 (0.87-1.23)	0.19	0.70	1.26 (0.84-1.88)	0.005	0.26	0.91 (0.63-1.31)	0.33	0.62	1.18 (0.84-1.65)	0.02	0.34	0.79 (0.52-1.20)	0.18	0.27
Korean	3	0.94 (0.78-1.13)	0.26	0.49	0.89 (0.72-1.11)	0.27	0.31	0.89 (0.54-1.48)	0.62	0.66	0.93 (0.75-1.15)	0.25	0.48	0.95 (0.58-1.57)	0.65	0.84
Iranians	2	1.57 (1.23-2.01)	0.34	**0.0003**	0.99 (0.68-1.45)	0.54	0.97	2.23 (1.42-3.51)	0.54	**0.0005**	1.38 (1.00-1.91)	0.78	0.05	2.23 (1.44-3.45)	0.41	**0.0003**
Others	3	1.11 (0.96-1.29)	0.88	0.16	1.22 (0.77-1.95)	0.04	0.40	1.24 (0.89-1.73)	0.67	0.20	1.19 (0.86-1.63)	0.16	0.30	1.13 (0.77-1.67)	0.21	0.53
Caucasian	3	0.95 (0.88-1.04)	0.39	0.28	0.93 (0.77-1.12)	0.12	0.44	0.96 (0.76-1.20)	0.97	0.71	0.93 (0.80-1.09)	0.19	0.36	0.98 (0.78-1.22)	0.87	0.83
**Cancer type**																
Liver cancer	8	1.12 (0.85-1.54)	<0.00001	0.46	1.14 (0.85-1.54)	0.0002	0.38	1.03 (0.55-1.92)	0.0005	0.93	1.15 (0.82-1.61)	<0.00001	0.43	0.99 (0.57-1.71)	0.002	0.97
Breast cancer	4	1.25 (0.99-1.57)	0.0002	0.06	1.15 (0.91-1.45)	0.04	0.24	1.43 (0.87-2.34)	0.009	0.16	1.24 (0.99-1.55)	0.03	0.06	1.30 (0.75-2.24)	0.002	0.35
Gastric cancer	4	1.03 (0.90-1.18)	0.49	0.66	0.98 (0.83-1.15)	0.54	0.79	1.21 (0.84-1.75)	0.66	0.31	1.01 (0.86-1.17)	0.53	0.95	1.22 (0.85-1.76)	0.66	0.28
Colorectal cancer	3	0.96 (0.82-1.13)	0.42	0.62	1.05 (0.87-1.26)	0.96	0.64	0.61 (0.23-1.62)	0.09	0.32	1.00 (0.84-1.20)	0.73	0.99	0.61 (0.23-1.58)	0.10	0.31
Lung cancer	2	0.96 (0.82-1.13)	0.83	0.64	0.98 (0.81-1.18)	0.60	0.84	0.88 (0.54-1.44)	0.92	0.61	0.97 (0.81-1.16)	0.68	0.74	0.87 (0.54-1.42)	0.84	0.59
Esophageal cancer	2	0.80 (0.66-0.98)	0.75	**0.03**	0.81 (0.63-1.04)	0.80	0.10	0.60 (0.33-1.08)	0.88	0.09	0.79 (0.62-1.00)	0.83	0.05	0.64 (0.36-1.14)	0.93	0.13
Others	8	1.11 (0.97-1.26)	0.05	0.13	1.27 (0.98-1.65)	<0.0001	0.07	0.99 (0.79-1.25)	0.80	0.93	1.12 (0.98-1.53)	0.0004	0.07	0.91 (0.72-1.16)	0.37	0.46

^a^Number of comparisons.

^b^P h value of Q-test for heterogeneity test. Random-effects model was used when P value for heterogeneity test <0.1; otherwise, fix-effects model was used.

^c^P value for significance, the cutoff point of statistical significance was set at p < 0.05, which was shown in boldface.

### Publication bias

**Figure 2:**
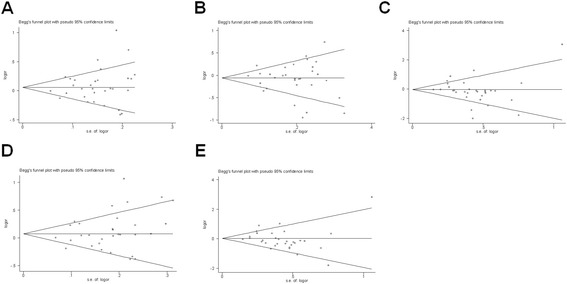
**Begg’s funnel plot for publication bias test. (A)** C versus T; **(B)** TC versus TT; **(C)** CC versus TT; **(D)** TC/CC versus TT (dominant) and **(E)** CC versus TC/TT (recessive). Each point represents a separate study for the indicated association. Log OR, natural logarithm of OR.

Begg’s funnel plot and Egger’s test were performed to assess the publication bias of included studies. Symmetrical funnel plots were obtained in all comparison models (Figure 2). Further, Egger’s test confirmed the absence of publication bias in all genetic models (*P* > 0.05).

### Sensitivity analysis

**Figure 3:**
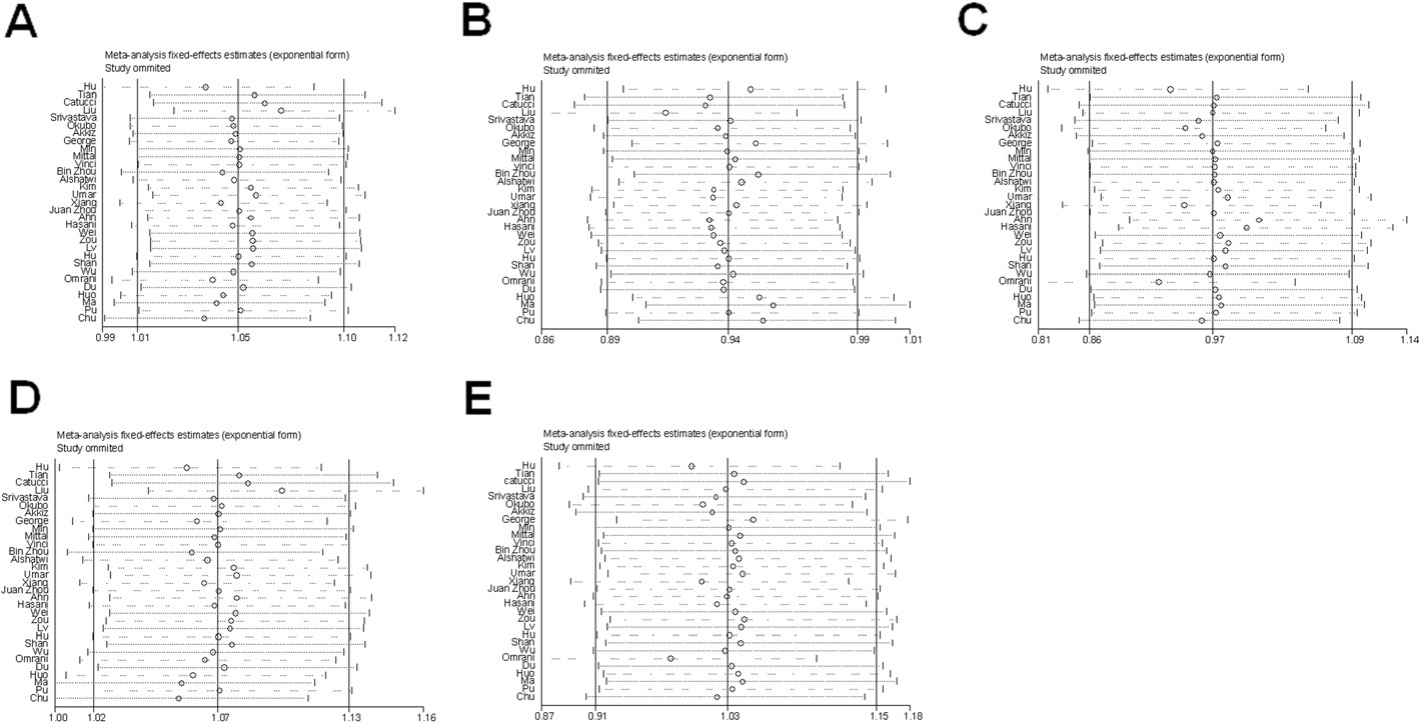
**Sensitivity analysis of the influence of a single study on the overall meta-analysis estimate. (A)** C versus T; **(B)** TC versus TT; **(C)** CC versus TT; **(D)** TC/CC versus TT (dominant) and **(E)** CC versus TC/TT (recessive). The solid lines correspond to the pooled OR and 95% CI. Circles and dashed lines correspond to the specific OR and 95% CI without omitted study.

Exclusion of one study at a time was performed to examine the influence of the individual data set to the pooled ORs. Analyses were performed using STATA by means of the metainf program. Results showed that the pooled OR estimates were similar with those of the excluded studies (Figure 3).

## Discussion

In the present study, hsa-mir-499 rs3746444 polymorphism and susceptibility to cancer was evaluated in different genetic models in 12799 cancer cases and 14507 controls. Significant association between rs3746444 polymorphism and susceptibility to cancer was identified in TC versus TT and TC/CC versus TT (dominant) models. Subgroup analysis absed on ethnicity revealed significantly increased risk in Asians (TC versus TT and TC/CC versus TT (dominant), but there was no significant association in Caucasians in all genetic models. This association was especially marked in Iranians (C versus T; CC versus TT and CC versus TC/TT (recessive) and a significant association was also identified in Chinese (TC versus TT) However, subgroup analysis based on cancer type revealed a decreased risk for esophageal cancer (C versus T).

Many studies investigated the role of SNPs presented in precursor and mature miRNAs, and their influences on susceptibility and progression of various diseases. miRNA-associated SNPs can have direct or indirect effects: 1) direct effects are produced by the SNPs in the pri-miRNA, pre-miRNA or mature miRNA that impair or enhance miRNA processing or function; 2) indirect effects are derived from the SNPs in miRNA promoters that affect transcription and SNPs in an mRNA that create or destroy a target site [71].

Genome-wide patterns of human polymorphisms in miRNAs and miRNA target sites in 3′ UTRs of mRNAs revealed that only ~10% of human pre-miRNAs have documented SNPs, and <1% of mature miRNAs have SNPs in the functional seed region [72]. hsa-mir-499 rs3746444 polymorphism is located in the seed region of the mature miR-499 sequence. This T > C polymorphism-resulted mismatch may affect target mRNA expression. Several case–control studies have investigated the association between rs3746444 polymorphism and risks of various cancers. However, the results were controversial.

Different from past studies, we analyzed the specific association among different ethnic groups throughout Asia. In this meta-analysis, we first found that rs3746444 polymorphism was associated with increased cancer risk mainly in Asian population, especially in Chinese and Iranians. It is important to notice that, although rs3746444 polymorphism contributes to cancer risk in Chinese and Iranians, the genetic models are different. The genetic variations between Chinese Han population and Arabian population may contribute to the differences. Interestingly, we also found that rs3746444 polymorphism showed a decreased risk in esophageal cancer. This suggests that potentially different mechanisms may underlie tumorgenesis in different pathological backgrounds and the environment variations.

It has been suggested that miRNAs may influence gene expression of ~30% of protein-coding genes by binding to the mRNA incompletely, and a miRNA may affect multiple target mRNAs involved in same pathophysiological progress [3,73]. This means that rs3746444 polymorphism may contribute to single gene function in certain disease model at molecular level. When taken together in epidemiological studies of populations, various effects of rs3746444 polymorphism on different genes may result in different associations with diseases at phenotype level.

Our meta-analysis pooled the largest numbers of cases and controls from included studies, which significantly increases the statistical power but some limitations should be considered. Firstly, lack of the consideration of combined genetic factors together with environmental exposures, while a more precise analysis needs to be conducted if more detailed data are available. Secondly, although sensitivity analysis showed that each study can not affect the overall results, some heterogeneity was evident in some of the comparisons. This may be due to different ethnicities and different pathology. And expanding the sample size may yield a better result. Thirdly, some unpublished data (such as negative report, not written in English and lack of studies in African population) may potentially influence the results of our meta-analysis. At last, different genotyping strategies may contribute to the bias in the analysis.

Recently several meta-analyses systematically reviewed the potential association of rs3746444 polymorphism with susceptibility to diseases [40–45,74–77]. However, the results were controversial across these meta-analyses. Such discrepancies in the results of these systematic reviews may due to different search periods, databases searched, inclusion and exclusion criteria. Our efforts to search the maximum range of databases yielded the most comprehensive studies in the similar time period especially the most recent publications [53,59]. Further, diagnosis of cancer is largely relied on confirmation by histology, imaging or pathology. Some of the other studies omitted this fundamental condition. Most importantly, deviations from Hardy-Weinberg equilibrium can inflate the chance of a false-positive association, and sensitivity analysis should be performed, pooling with and without studies not in HWE, to test the robustness of the results [78,79]. Only few of other studies performed sensitivity analysis, which may limit the reliability of their conclusions. Although some studies claimed that their studies were in HWE, a careful examination of the studies revealed that the data did not reach HWE. In conclusion, in our study, relevant literatures selected from broad databases with stringent standards would be expected to provide a more reliable conclusion.

## Conclusions

In summary, though with limitations, our meta-analysis suggested that hsa-mir-499 rs3746444 T > C polymorphism is associated with the risk of cancer, especially in Asians, mainly in Chinese and Iranians. However, rs3746444 T > C polymorphism is negatively associated with the risk of esophageal cancer. Larger studies from different ethnic groups and studying different types of cancers with detailed information are needed to further clarify the association between rs3746444 polymorphism and cancer risk.

## Additional files

Additional file 1:
**The PRISMA 2009 Checklist.**
Additional file 2:
**Forest plot of ORs for the association of hsa-miR-499 rs3746444 T>C polymorphism with cancer risk in different situations.** **Figure S1.** Forest plot of ORs for the association of hsa-miR-499 rs3746444 T>C polymorphism with cancer risk is illustrated by ethnicity. (A) C versus T; (B) TC versus TT; (C) CC versus TT; (D) TC/CC versus TT (dominant) and (E) CC versus TC/TT (recessive). **Figure S2.** Forest plot of ORs for the association of hsa-miR-499 rs3746444 T>C polymorphism with cancer risk is illustrated by cancer type. (A) C versus T; (B) TC versus TT; (C) CC versus TT; (D) TC/CC versus TT (dominant) and (E) CC versus TC/TT (recessive). **Figure S3.** Forest plot of ORs for the association of hsa-miR-499 rs3746444 T>C polymorphism with cancer risk is illustrated by country in Asia. (A) C versus T; (B) TC versus TT; (C) CC versus TT; (D) TC/CC versus TT (dominant) and (E) CC versus TC/TT (recessive).
