# Supplementary material for: Meta-analysis of Hsa-mir-499 polymorphism (rs3746444) for cancer risk: evidence from 31 case-control studies
Source: BMC Med Genet. 2014 Nov 30;15:126. doi: 10.1186/s12881-014-0126-1 (PMC4411927; doi:10.1186/s12881-014-0126-1)
Supplement: Additional file 2: — Forest plot of ORs for the association of hsa-miR-499 rs3746444 T>C polymorphism with cancer risk in different situations. Figure S1. Forest plot of ORs for the association of hsa-miR-499 rs3746444 T>C polymorphism with cancer risk is illustrated by ethnicity. (A) C versus T; (B) TC versus TT; (C) CC versus TT; (D) TC/CC versus TT (dominant) and (E) CC versus TC/TT (recessive). Figure S2. Forest plot of ORs for the association of hsa-miR-499 rs3746444 T>C polymorphism with cancer risk is illustrated by cancer type. (A) C versus T; (B) TC versus TT; (C) CC versus TT; (D) TC/CC versus TT (dominant) and (E) CC versus TC/TT (recessive). Figure S3. Forest plot of ORs for the association of hsa-miR-499 rs3746444 T>C polymorphism with cancer risk is illustrated by country in Asia. (A) C versus T; (B) TC versus TT; (C) CC versus TT; (D) TC/CC versus TT (dominant) and (E) CC versus TC/TT (recessive). [file 12881_2014_126_MOESM2_ESM.doc]

**A**


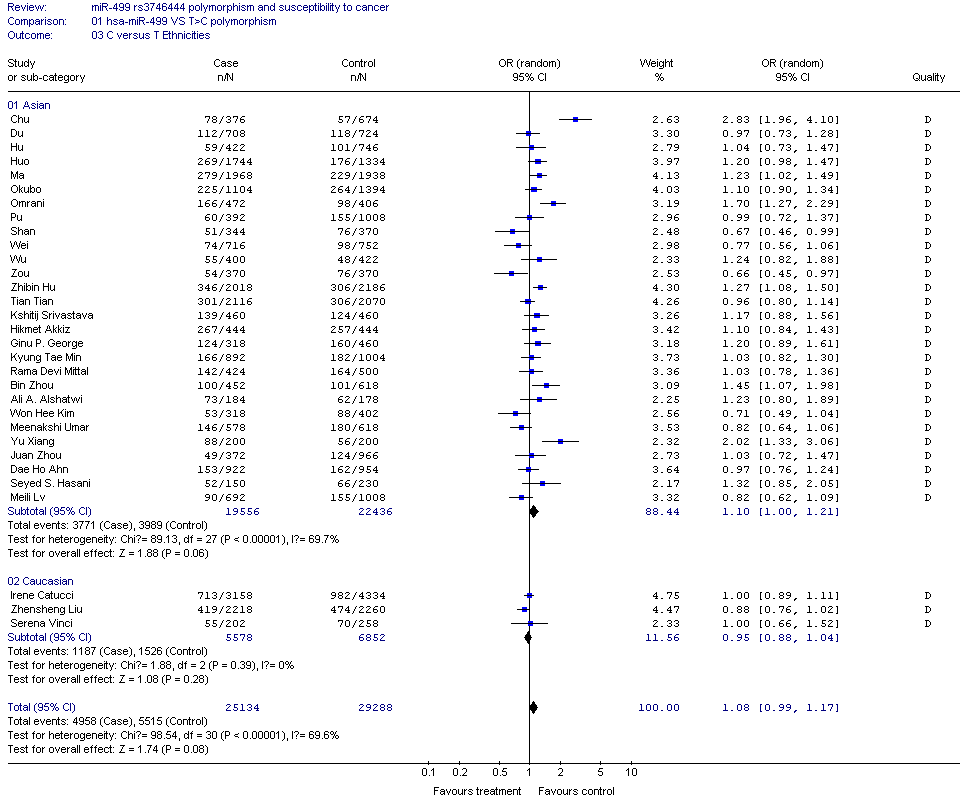


**B**

**
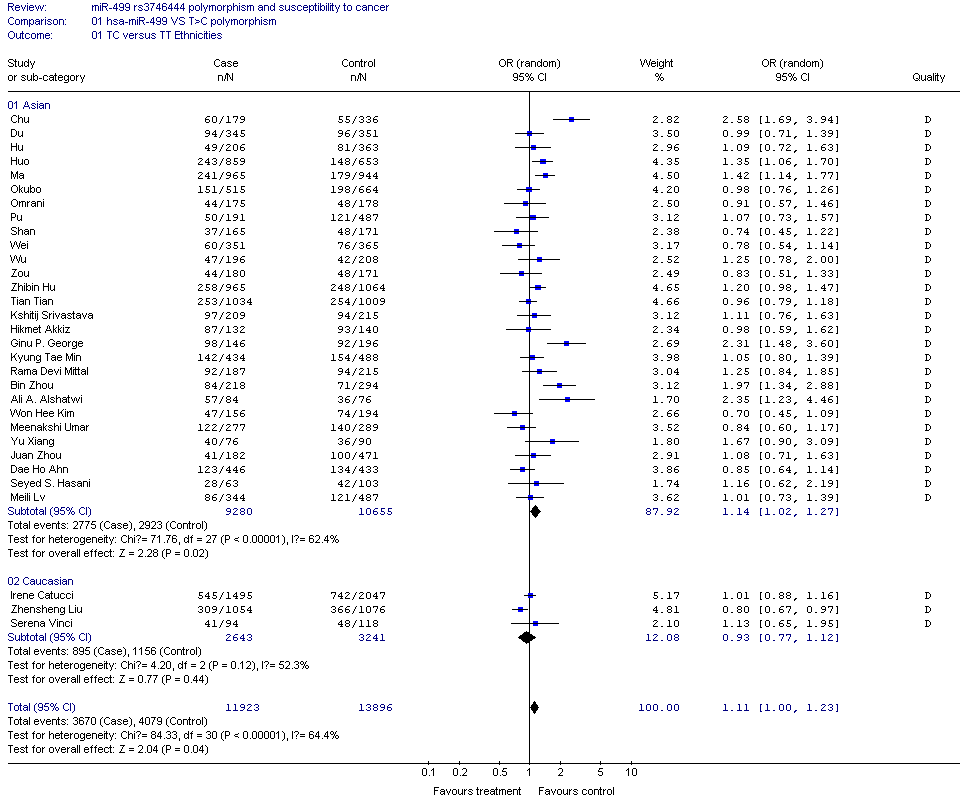
**

**C**

**
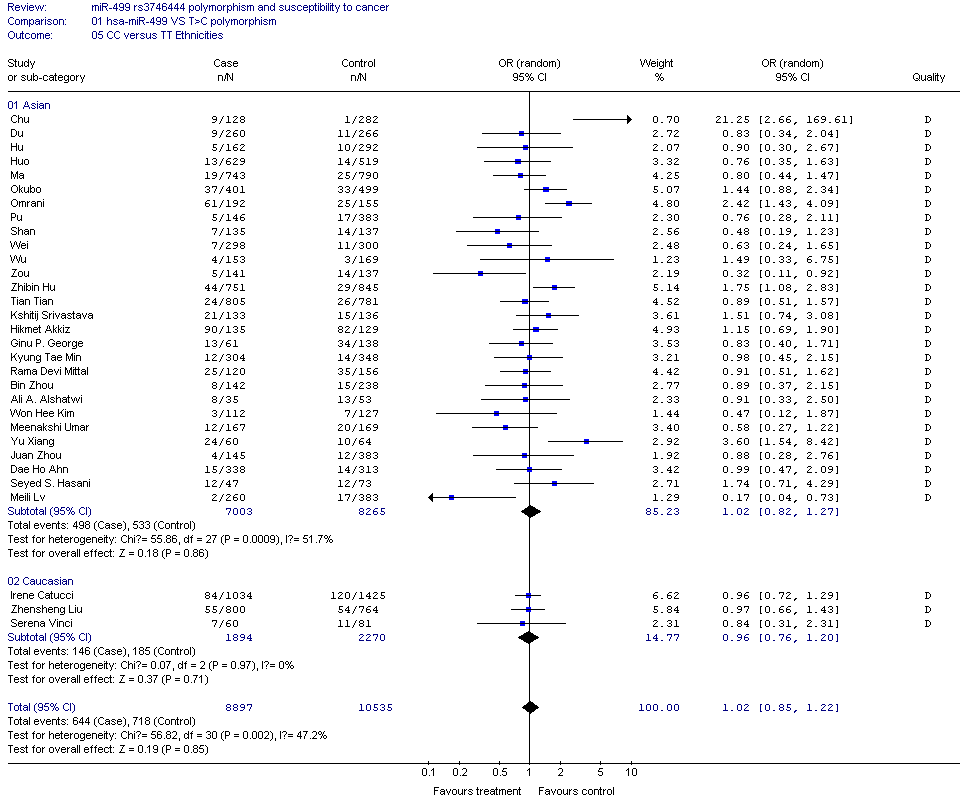
**

**D**

**
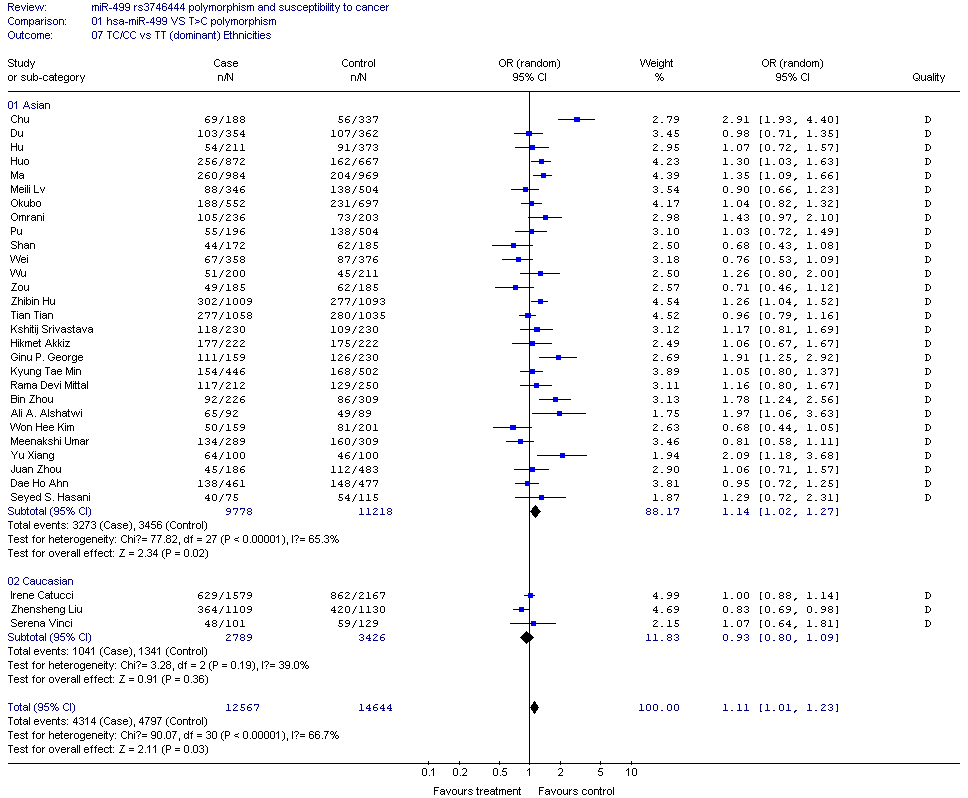
**

**E**

**
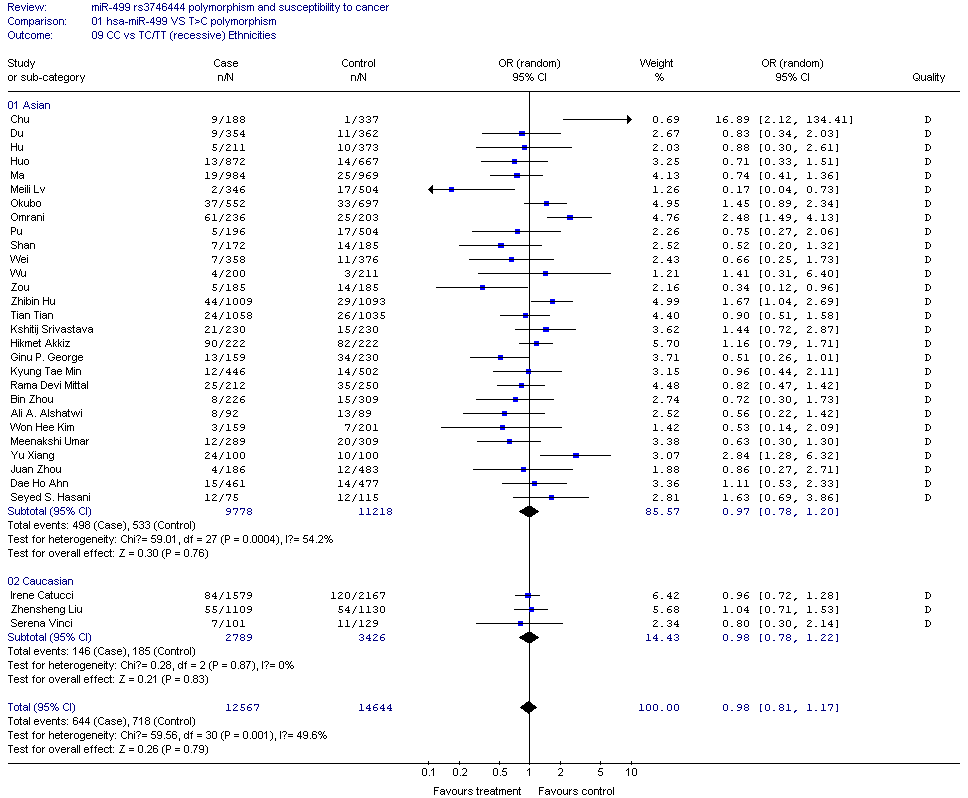
**

**Supplemental Figure S1. Forest plot of ORs for the association of hsa-miR-499 rs3746444 T>C polymorphism with cancer risk is illustrated by ethnicity.** (A) C versus T; (B) TC versus TT; (C) CC versus TT; (D) TC/CC versus TT (dominant) and (E) CC versus TC/TT (recessive).

**A**

**
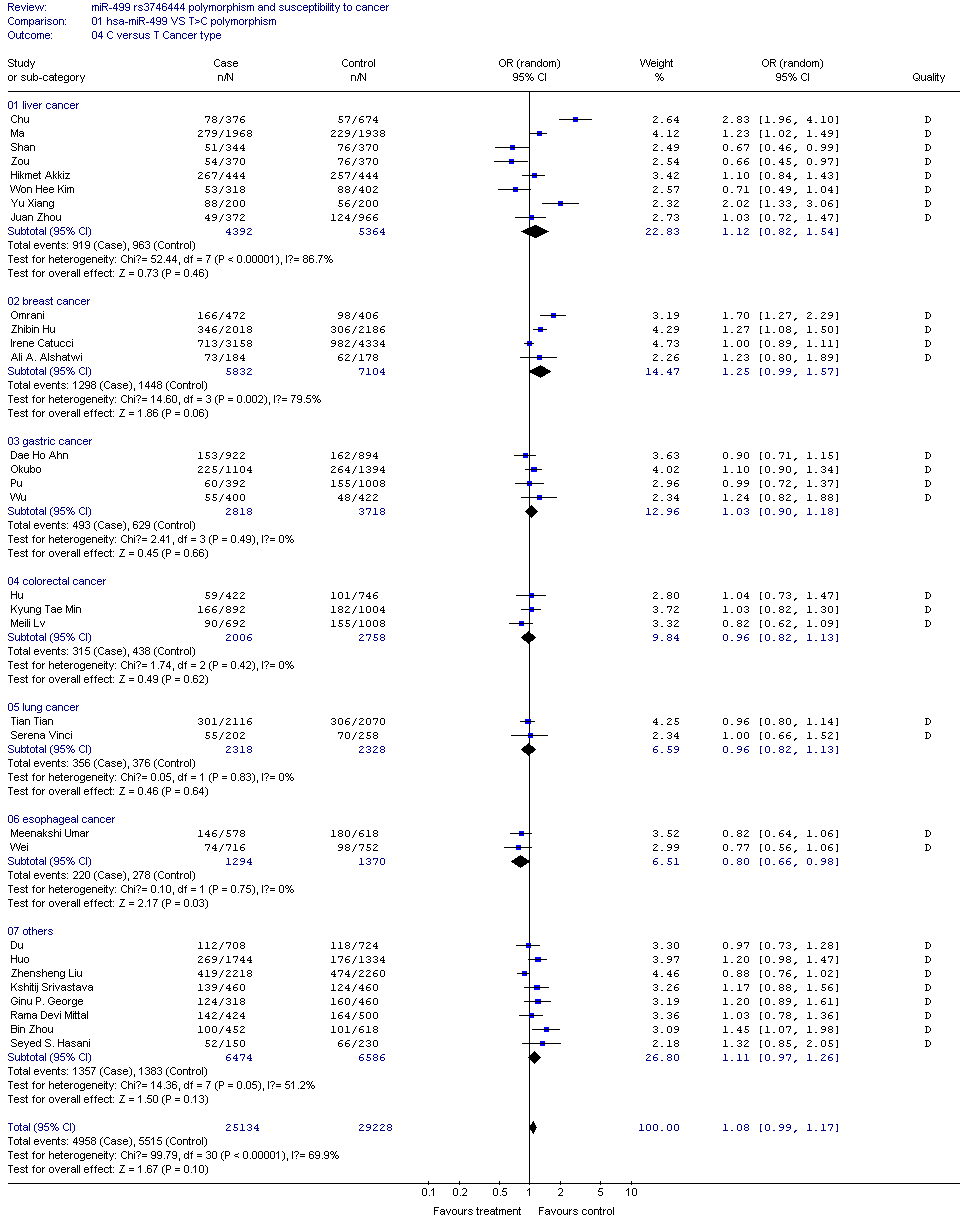
**

**B**

**
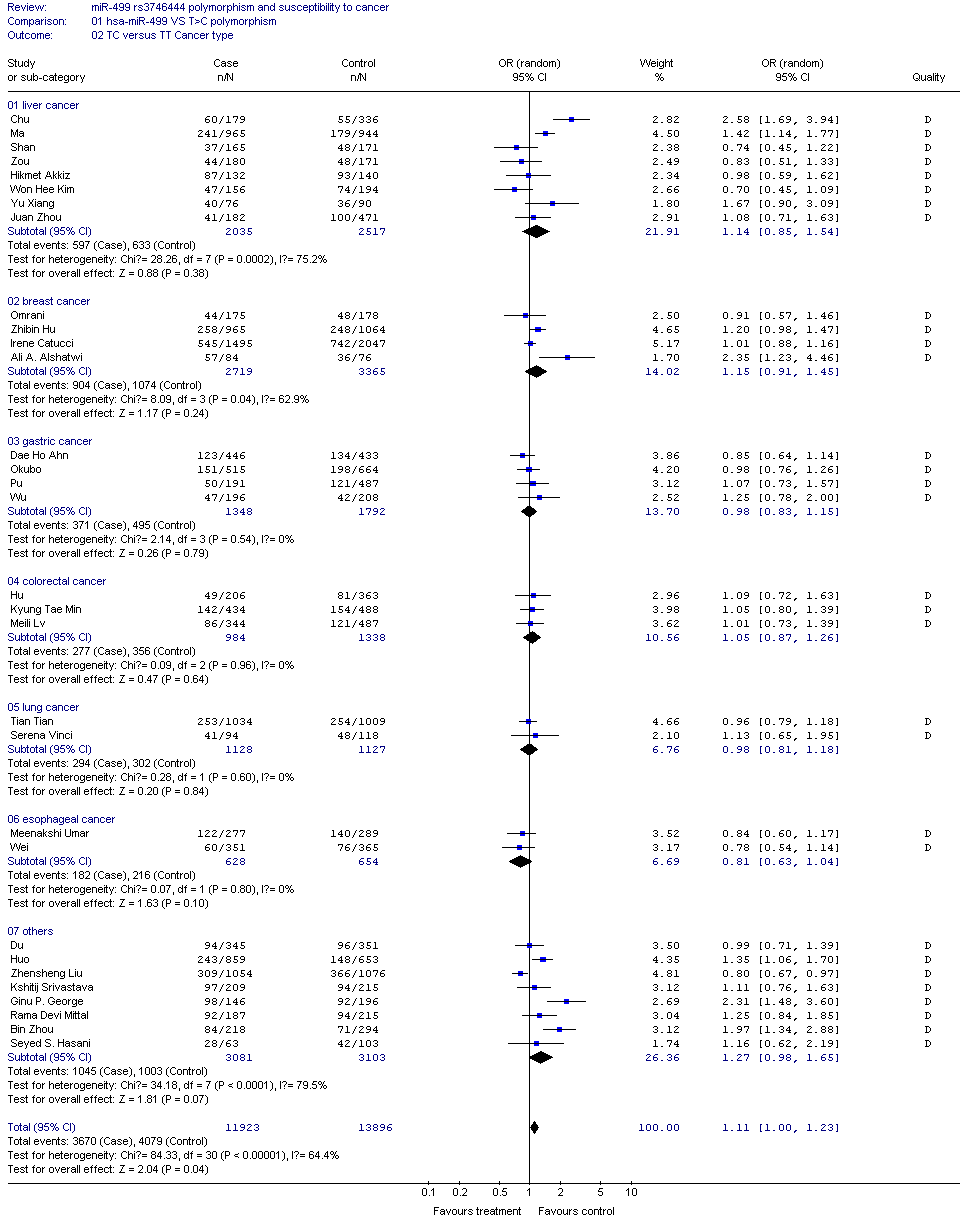
**

**C**

**
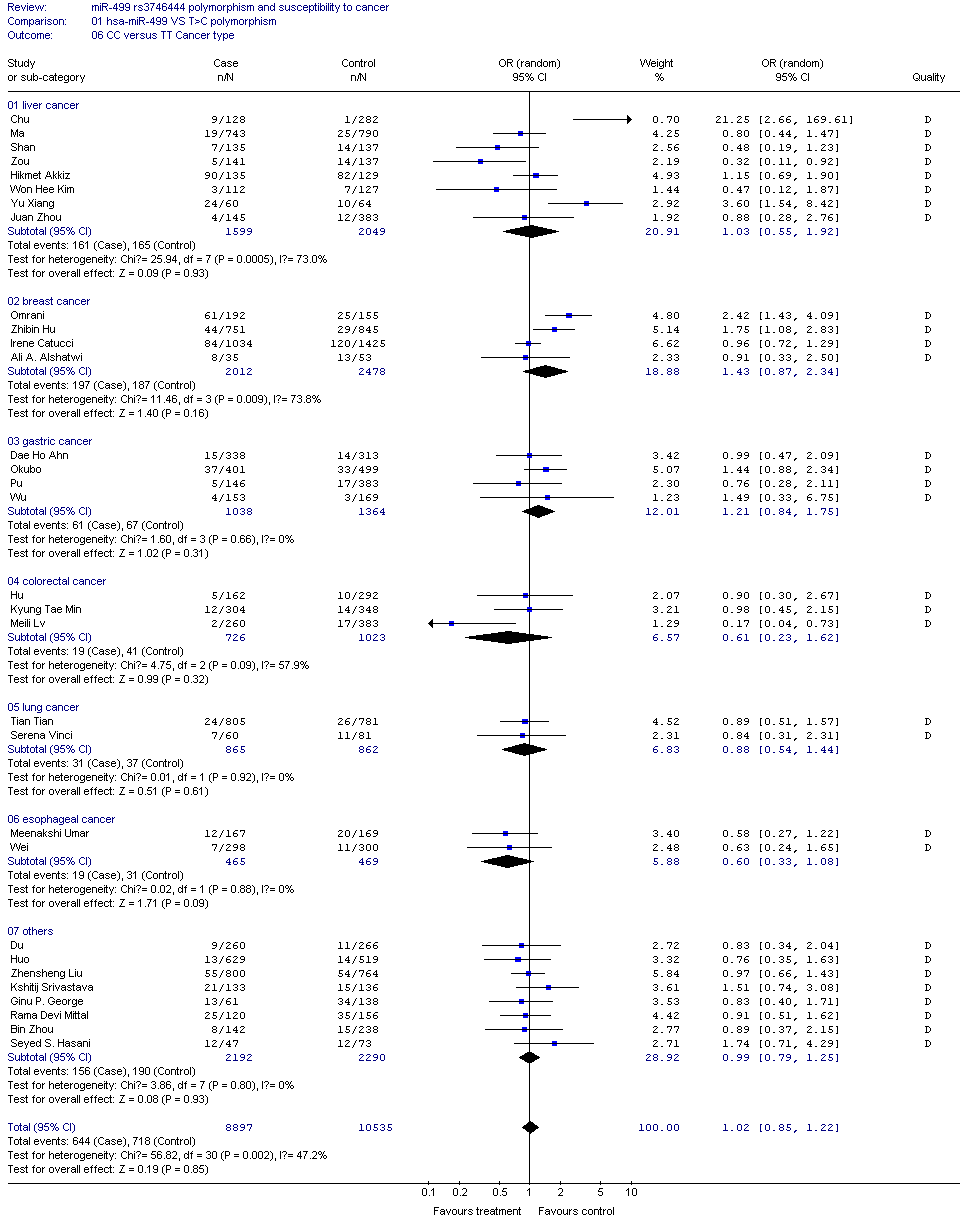
**

**D**

**
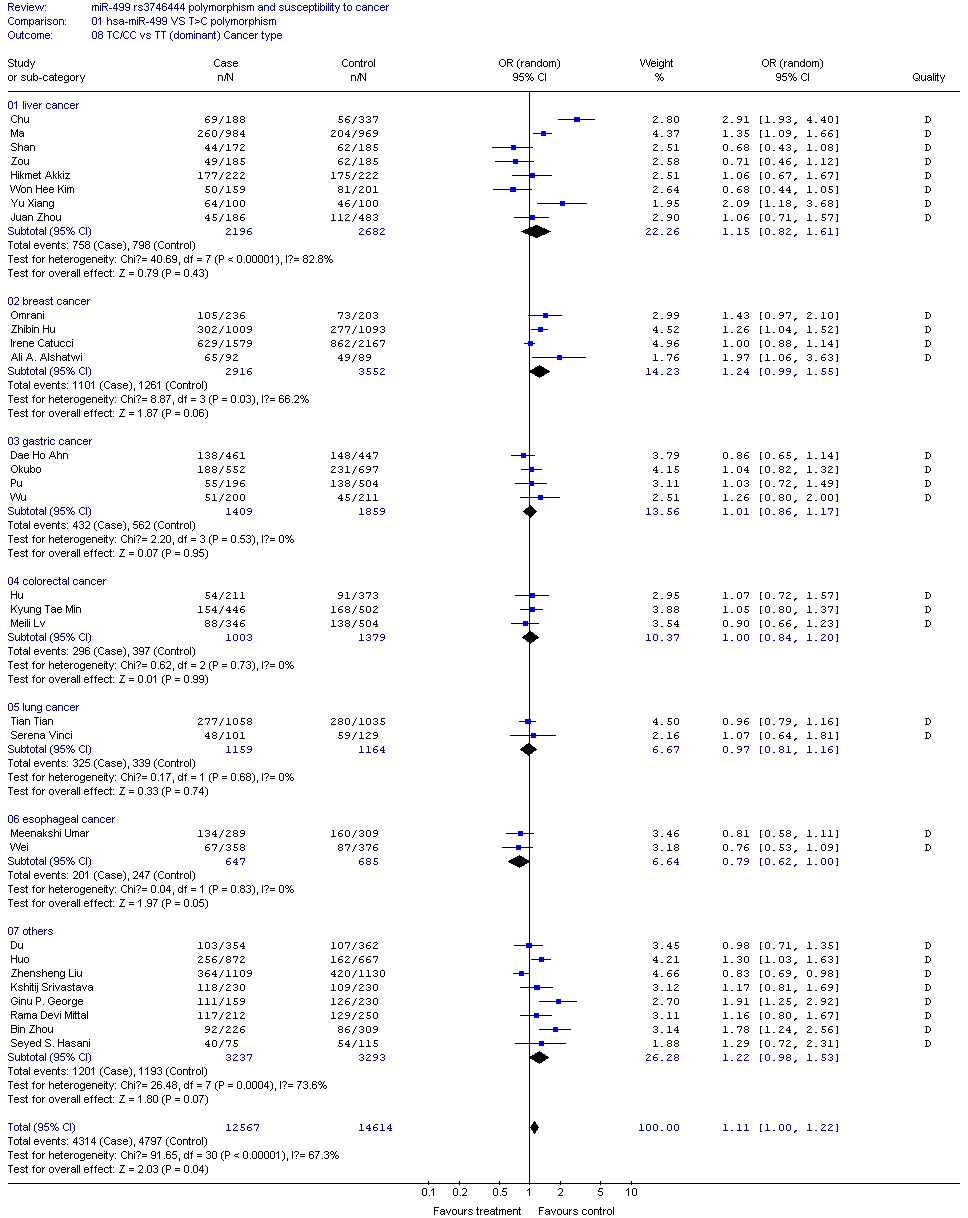
**

**E**

**
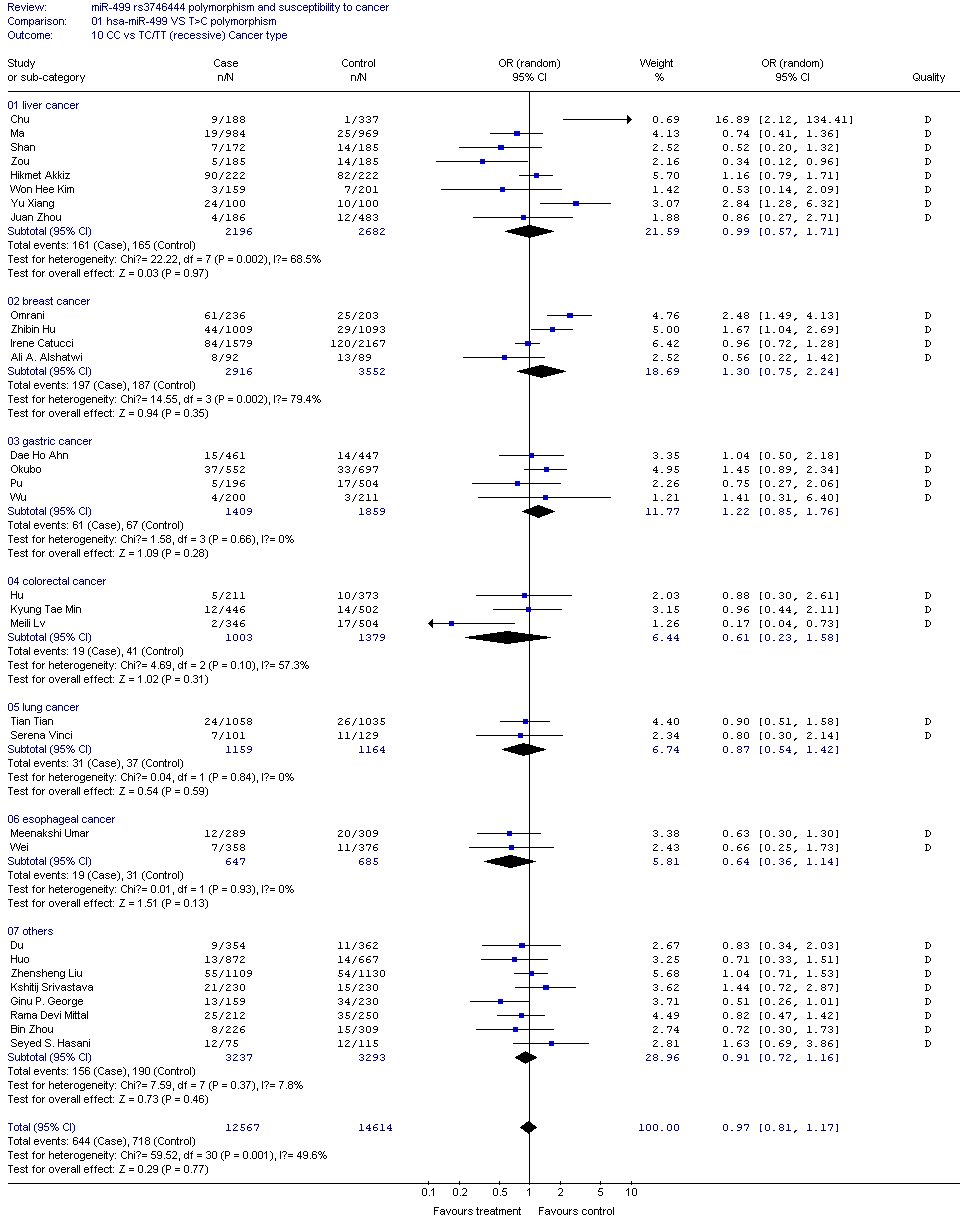
**

**Supplemental Figure S2. Forest plot of ORs for the association of hsa-miR-499 rs3746444 T>C polymorphism with cancer risk is illustrated by cancer type.** (A) C versus T; (B) TC versus TT; (C) CC versus TT; (D) TC/CC versus TT (dominant) and (E) CC versus TC/TT (recessive).

**A
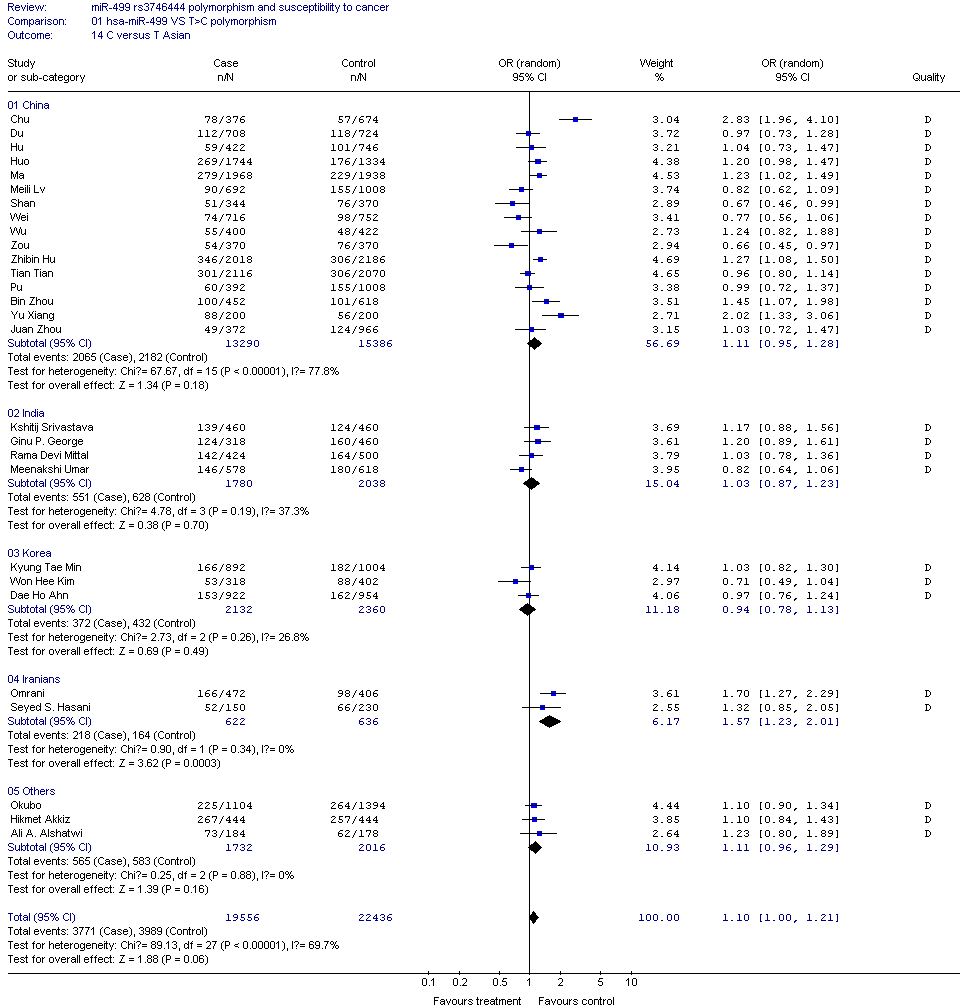
**

**B**

**
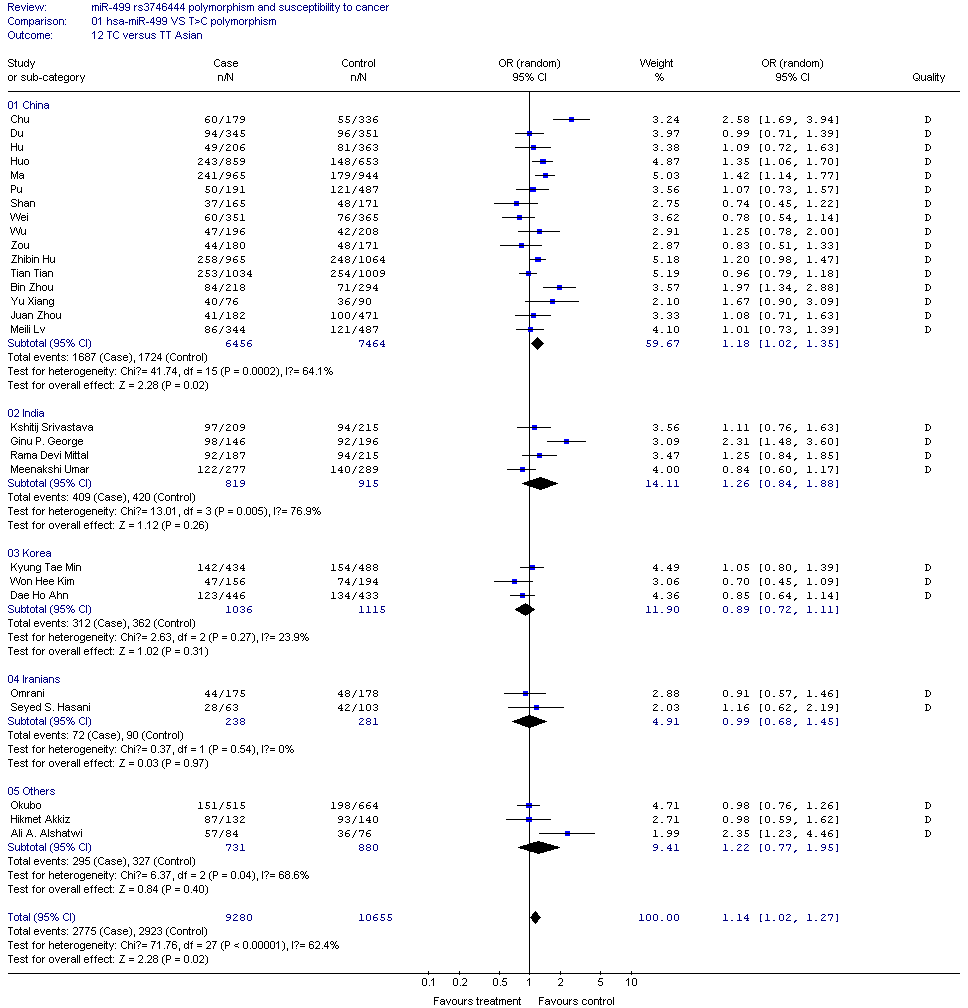
**

**C**

**
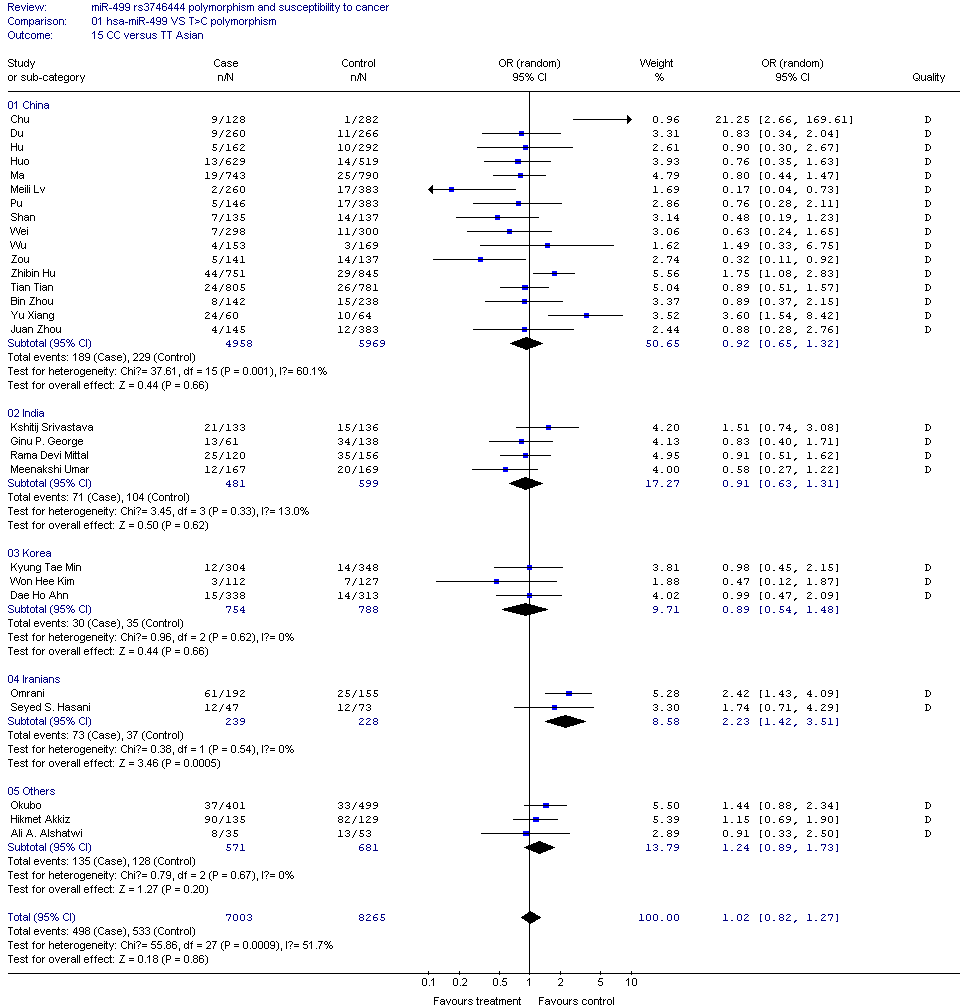
**

**D**

**
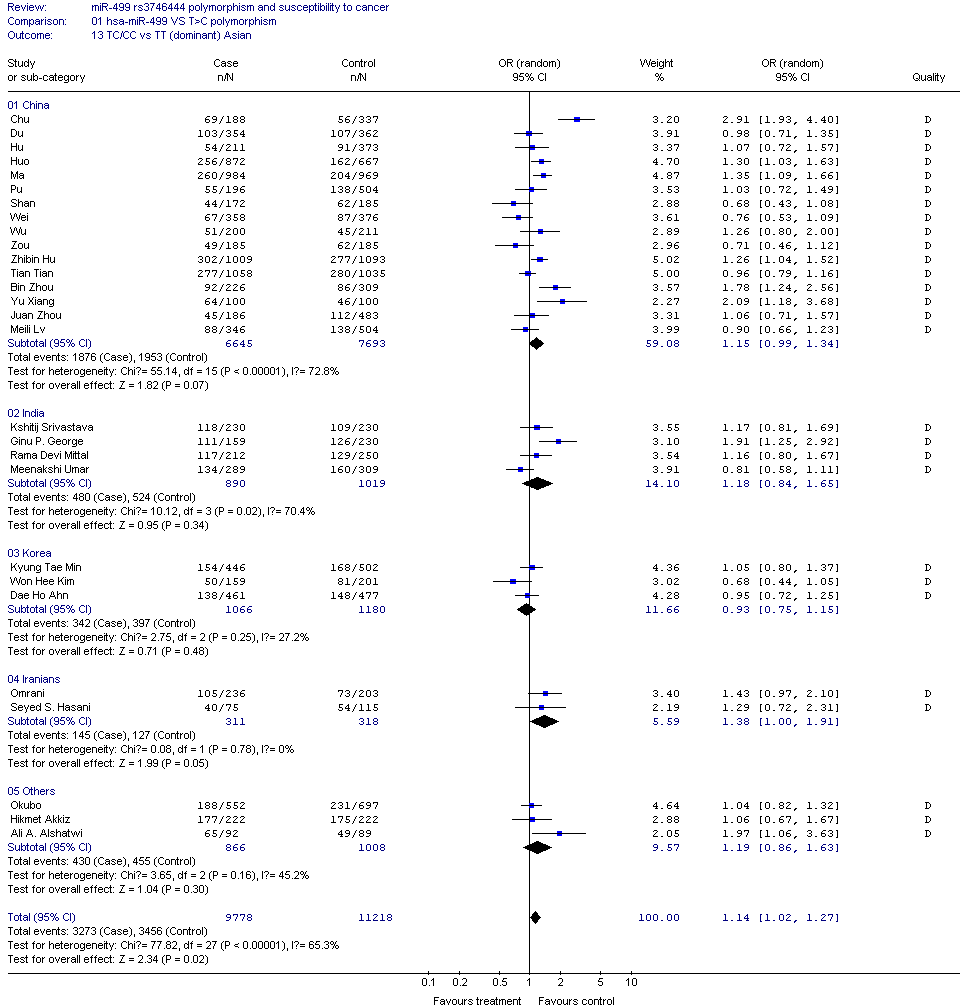
**

**E**

**
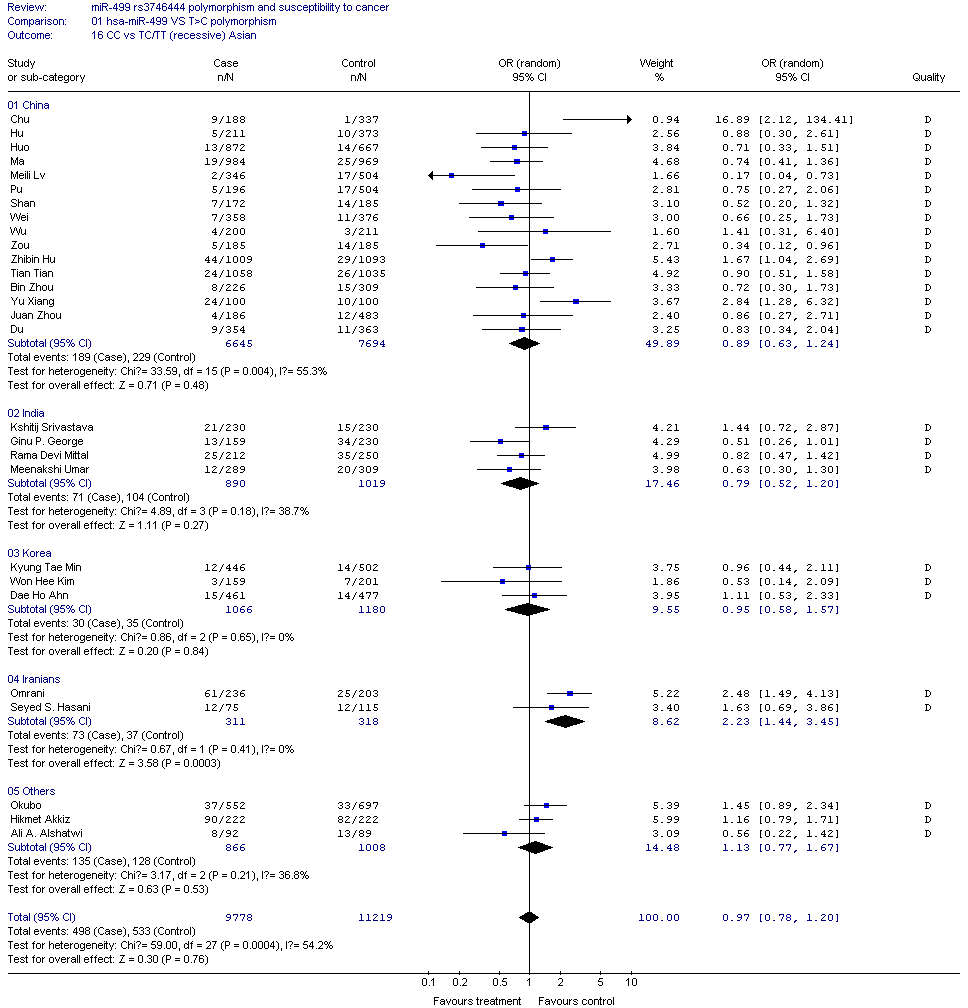
**

**Supplemental Figure S3. Forest plot of ORs for the association of hsa-miR-499 rs3746444 T>C polymorphism with cancer risk is illustrated by country in Asia.** (A) C versus T; (B) TC versus TT; (C) CC versus TT; (D) TC/CC versus TT (dominant) and (E) CC versus TC/TT (recessive).
